# Dissipatively coupled waveguide networks for coherent diffusive photonics

**DOI:** 10.1038/s41467-017-02048-4

**Published:** 2017-12-04

**Authors:** Sebabrata Mukherjee, Dmitri Mogilevtsev, Gregory Ya. Slepyan, Thomas H. Doherty, Robert R. Thomson, Natalia Korolkova

**Affiliations:** 1 Scottish Universities Physics Alliance (SUPA), Institute of Photonics and Quantum Sciences, School of Engineering & Physical Sciences, Heriot-Watt University, Edinburgh, EH14 4AS UK; 2 Institute of Physics, Belarus National Academy of Sciences, F. Skarina Ave. 68, Minsk, 220072 Belarus; 3 Department of Physical Electronics, School of Electrical Engineering, Faculty of Engineering, Tel Aviv University, Tel Aviv, 69978 Israel; 4 School of Physics and Astronomy, University of St Andrews, North Haugh, St Andrews, KY16 9SS UK

## Abstract

A photonic circuit is generally described as a structure in which light propagates by unitary exchange and transfers reversibly between channels. In contrast, the term ‘diffusive’ is more akin to a chaotic propagation in scattering media, where light is driven out of coherence towards a thermal mixture. Based on the dynamics of open quantum systems, the combination of these two opposites can result in novel techniques for coherent light control. The crucial feature of these photonic structures is dissipative coupling between modes, via an interaction with a common reservoir. Here, we demonstrate experimentally that such systems can perform optical equalisation to smooth multimode light, or act as a distributor, guiding it into selected channels. Quantum thermodynamically, these systems can act as catalytic coherent reservoirs by performing perfect non-Landauer erasure. For lattice structures, localised stationary states can be supported in the continuum, similar to compacton-like states in conventional flat-band lattices.

## Abstract (web summary)

Diffusive light propagation represents a valuable additional tool for integrated photonic technologies. As an example, here the authors experimentally demonstrate optical equalisation of coherent light propagating in a femtosecond laser written circuit which simulates a dissipatively-coupled quantum chain.

## Introduction

The engineering of dissipation to a common reservoir generates a vast array of novel structures for photonic application and quantum simulation. It has already been shown that the coupling of a number of quantum systems to the same reservoir gives rise to a decoherence-free subspace of Hilbert space^1^. Moreover, the evolution of an initial state towards this decoherence-free subspace is able to preserve and even create entanglement^2–5^. The careful engineering of loss can lead to coherence preservation^6–8^, deterministic creation of non-classical states^9–11^ and serve as a tool for quantum computation^12, 13^. Networks of dissipatively coupled systems were studied that can support topologically protected states^14–16^. Recently, optical setups were also used to study phenomena induced by engineered losses^17–19^.

Arrays of evanescently coupled optical waveguides is an excellent experimental platform to investigate a wide variety of semi-classical and quantum phenomena ranging from robust topological edge states^20, 21^ to quantum walks of correlated photons^22^. Precise control in waveguide fabrication allows access to a desired Hamiltonian and the ability to probe the evolution of a specific initial state. Waveguide arrays with controllable loss and/or gain are also used to study various effects associated with non-Hermitian physics^23^. In recent years, development of the femtosecond laser writing technique^24^ facilitated the fabrication of optical waveguides and waveguide-based devices with three-dimensional geometry enabling the demonstration of intriguing phenomena known from condensed matter and quantum physics^25^.

Using the platform of integrated waveguide networks, here we propose that light can flow diffusively while remaining coherent and even entangled in a system of bosonic modes coupled to common reservoirs. In the experiment, performed using classical input states, we observed coherent diffusive equalisation in dissipatively coupled waveguide arrays. Coherent diffusive light propagation opens new vistas for photonic applications, such as directional light distribution and diffusive coherence-preserving equalisation. In other words, we demonstrate that the aforementioned phenomena can be realised in the network of coupled integrated waveguides suggesting an exciting area in optical technologies, coherent diffusive photonics.

## Results

### Theoretical background

**Fig. 1:**
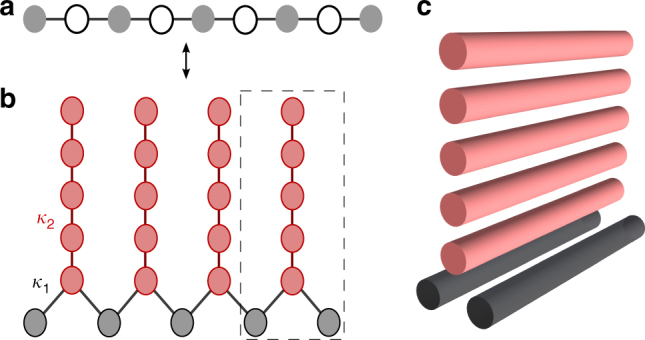
One-dimensional diffusive photonic circuit. **a** Dissipatively coupled chain of the single mode waveguides. Every second waveguide (empty circles) is lossy and serves as a reservoir. The waveguides indicated by filled circles exhibit low loss and couple dissipatively via the auxiliary waveguides only. **b** Experimental realisation of the chain in **a** using an integrated optical circuit. The lossy sites are implemented using auxiliary arrangements of coupled waveguides. Here, *κ*_1_ is the coupling between chain modes and lossy sites and *κ*_2_ is the coupling between the waveguides forming the reservoirs. **c** The three-dimensional geometry of the elementary diffusive circuit indicated by the dotted rectangle in **b**

The coherent diffusive photonic circuits considered in this article are described by the following generic quantum master equation:

1 $$\frac{{\rm{d}}}{{{\rm{d}}t}}\rho = \mathop {\sum}\limits_{j = 1}^N \gamma _j\left( {2A_j\rho A_j^\dagger - \rho A_j^\dagger A_j - A_j^\dagger A_j\rho } \right),$$

where *ρ*(*t*) is the density matrix, *A*_*j*_ denote the Lindblad operators for mode *j* and *γ*_*j*_ are the relaxation rates into corresponding reservoirs (see Supplementary Note 1 for more detail). In the experiments, we use femtosecond laser inscribed^24^ arrays of coupled optical waveguides, where the propagation of the light can mimic the time evolution described by specific Hamiltonians^25^. The relaxation rates (*γ*_*j*_) then describe the coherence diffusion rate between neighbouring waveguides. Coupling to common reservoirs is realised by mutually coupling each pair of waveguides to a linear arrangement of further waveguides^26^ (Fig. 1).

Let us start with a simple example of 1D dissipatively coupled chain (DCC) with *A*_*j*_ = *a*_*j*_ − *a*_*j*+1_, where *a*_*j*_ $( {a_j^\dagger })$ is the bosonic annihilation (creation) operator, $a_j\left| {\alpha _j} \right\rangle = \alpha _j\left| {\alpha _j} \right\rangle$. Equation (1) can then be recast in terms of coherent amplitudes *α*_*j*_ (Supplementary Note 1):

2 $$\frac{{\rm{d}}}{{{\rm{d}}t}}\alpha _k = - \left( {\gamma _k + \gamma _{k - 1}} \right)\alpha _k + \gamma _k\alpha _{k + 1} + \gamma _{k - 1}\alpha _{k - 1}.$$

Equation (2) formally coincide with the equations of a time-dependent classical random walk in one dimension, the discrete analogue of diffusion and heat transport dynamics. However, there are no classical probabilities in Eqs. (1) and (2), with the amplitudes *α*_*j*_ being complex. While the light flows diffusively, like heat, its coherence is maintained: off-diagonal elements in the Fock-state basis do not decay. For this to be the case, a fundamental role is played by the collective symmetrical superposition of all modes, characterised by a sum of modal operators:

3 $$A_{{\mathrm{sum}}} = \mathop {\sum}\limits_{j = 1}^{N + 1} \frac{{a_j}}{{\sqrt {N + 1} }}.$$

If a state is not symmetrical over all modes, it follows from Eqs. (1) and (2) that it will asymptotically decay to the vacuum state. Therefore, any state represented by a combination of operators *A*_sum_ and $A_{{\mathrm{sum}}}^\dagger$ is conserved by the dynamics of Eq. (3). These states can be quite diverse in nature, from highly non-classical to Gibbs states (see Supplementary Note 3). Furthermore, a stationary state can also be entangled: for a single photon in the DCC, the state $A_{{\mathrm{sum}}}^\dagger \mathop {\prod}\limits_{\forall j} \left| 0 \right\rangle _j$ is stationary (see ref. ^27^ for details).

**Fig. 2:**
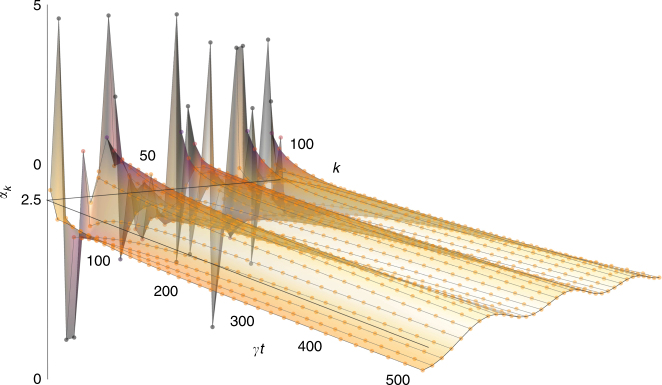
Diffusive equalisation in a one-dimensional chain. Simulation showing optical amplitude equalisation for *k* = 100 coherent states, propagating through a dissipatively coupled chain with equal coupling, *γ*_*j*_ = *γ*. The dynamics follow Eq. (1), where an initially random distribution of coherent state amplitudes are smoothed after a long effective time (*γt*). Darker data points indicate a larger deviation from the mean amplitude, *α* = 2.5, emphasised by an equivalently coloured surface. Data points corresponding to each individual oscillator are connected

Consider an initialisation with all modal oscillators in coherent states. Eq. (1) shows that this will evolve into a product of coherent states with equal and averaged amplitudes, $\mathop {\prod}\limits_{\forall j} \left| {\alpha _{{\mathrm{sum}}}} \right\rangle _j$ where $\alpha _{{\mathrm{sum}}} = \mathop {\sum}\limits_j \alpha _j{\mathrm{/}}\left( {N + 1} \right)$. This feature of the diffusive, yet coherent 1D circuit, opens the possibility to realise an optical equaliser, suppressing both intensity and phase fluctuations in multimode fields. The equaliser performance is illustrated in Fig. 2, where it is shown how the DCC can completely smooth any arbitrary zero-mean variations of the input.

### Photonic implementation

**Fig. 3:**
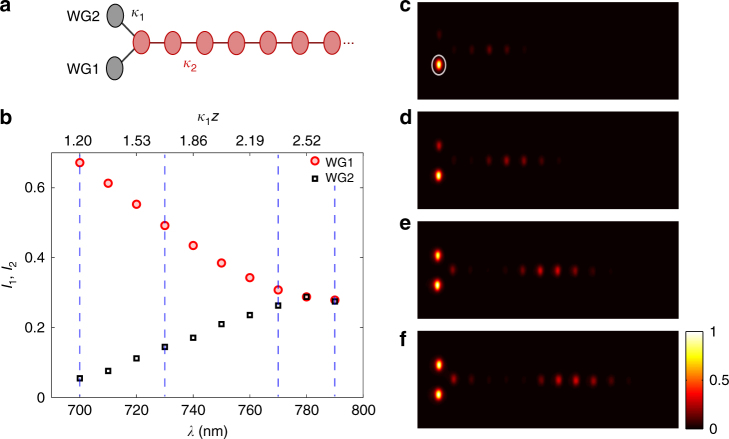
Coherent diffusive equalisation in a dissipatively coupled waveguide pair. **a** Sketch illustrating the photonic implementation of two sites coupled via a reservoir (same as Fig. 1c). Here, WG1 and WG2 are two waveguides comprising the chain and the array of coupled waveguides, indicated by pink circles, acts as the reservoir. **b** The equalisation effect: WG1 is excited initially with a coherent input and the light intensities (*I*_1,2_) at WG 1, 2 are measured after a propagation of *z* = 30 mm. The coherent diffusive evolution distributes the input light equally between WG1 and WG2. In this implementation, the effective propagation time *γt* of Eq. (1) and Fig. 2 translates into propagation distance *z* along the grey waveguides in Fig. 1 c. For a fixed sample length *z*, the dynamics can then be best assessed by monitoring the output intensity as a function of the wavelength as shown in the graph (see text and Supplementary Notes 1 and 2 for details). This effectively corresponds to changing *γt* ↔ *κ*_1_ *z*. **c**–**f**, Output intensity distributions for four different wavelengths (700, 730, 770 and 790 nm, respectively), indicated by the vertical dashed lines in **b**

**Fig. 4:**
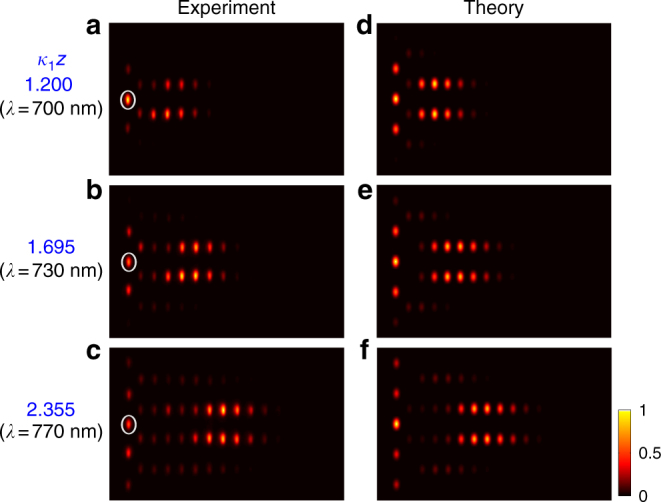
Coherent diffusive equalisation in a dissipatively coupled waveguide array. **a**–**c** Experimentally measured intensity distributions for three different values of *γt* ↔ *κ*_1_ *z*. Here, the maximum propagation distance of the device is *z* = 30 mm, *κ*_1_ is tuned by varying the wavelength (*λ*) of incident light, as in Fig. 3. The central waveguide of the chain, indicated by the white circle, was excited at the input for all measurements. In this device, the waveguides in the chain are coupled via identical reservoirs, each containing twenty coupled waveguides. **d**–**f** Numerically calculated intensity distributions corresponding to **a**–**c** respectively

In order to realise engineered dissipative coupling in integrated waveguides in accordance with Eq. (1), one must be able to adiabatically eliminate lossy sites (Fig. 1a) from the system dynamics. This results in the fine-tuning of the evanescent coupling parameters *κ*_1,2_, where the coupling between chain modes and lossy sites, *κ*_1_, must be considerably smaller than intra-reservoir couplings, *κ*_2_. For the particular design of Fig. 1c, it appears sufficient to have *κ*_1_/*κ*_2_ ≈ 0.5 (see Supplementary Fig. 1). The same ratio *κ*_1_/*κ*_2_ should hold for all *γt* (equivalently, *κ*_1_ *z* in waveguide implementation, where *z* is the propagation distance along the chain waveguides). The length of the DCC is not a prohibitive parameter and the collective behaviour, the coherent diffusive dynamics, can be established for merely two coupled bosonic modes. The effect of coherent optical equalisation can thus be readily achieved in the elementary circuit of Fig. 1c. In the experiment, a 30-mm-long elementary circuit with 20 waveguides in the reservoir was fabricated (Methods section) and the output intensity distribution was measured as a function of the wavelength, *λ*, of incident light. It should be mentioned that both *κ*_1,2_ vary linearly in the wavelength range of interest without affecting *κ*_1_/*κ*_2_ significantly, and hence, wavelength tuning enables us to observe the dynamics as the effective analogous time, *κ*_1_(*λ*)*z*, is tuned in this case (Supplementary Note 2). Figure 3 depicts the corresponding experimental results, clearly demonstrating equalisation of the input coherent signal. In the next step, we fabricated a chain of five waveguides, coupled via similar reservoirs, and demonstrate the coherent diffusive equalising. We excited the central waveguide of the chain at the input and measured the intensity distribution after a propagation of *z* = 30 mm. Figure 4 shows the output intensities for three different values of *κ*_1_ *z*. These experimental results are in good agreement with the numerically calculated output intensity distributions. Figure 4 shows dynamically how the equalisation unfolds.

### Two-dimensional diffusive circuits

**Fig. 5:**
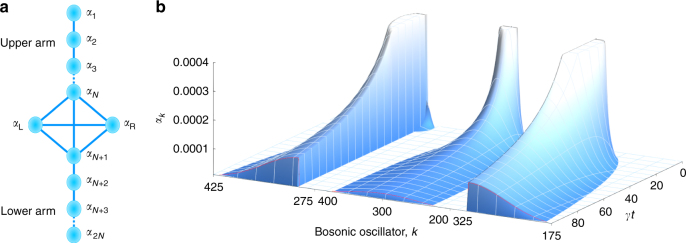
Excitation distribution in a two-arm dissipative structure. **a** Illustration of two dissipatively coupled linear chains interacting via a pair of control modes. There are *N* waveguides in each arm. **b** The coherent and guided transportation of light for three different states of the control modes. For these simulations, the number of waveguides in each arm is *N* = 300, with only mode *α*_*N*=300_ being excited initially. In the leftmost distribution, the control modes, *α*_L_ and *α*_R_, have opposite phasing and light is guided into the lower arm. In the central scenario, *α*_L_ is excited and light equalises over the entire structure. In the rightmost case, the control modes are excited with equal amplitude and phase, confining light to the upper arm only

When the linear arrangement of modes in the DCC is extended to further dimensions, a large vista of applications becomes accessible. These range from re-routing photonic devices to simulators of many-body quantum systems. Figure 5a outlines a photonic circuit for which the excitation of two control modes can dissipatively direct a coherent flow of light (Fig. 5b). This Quantum Distributor comprises two linear DCC, connected by mutual interaction to the pair of control modes (Supplementary Note 4). Here, the control modes perform the distribution catalytically, their coherence being conserved.

Another simple DCC structure comprises two linear chains placed parallel with dissipative connections between each neighbouring mode (Supplementary Fig. 2). This arrangement gives rise to the localisation of signals which, unusually, is not born of defects (Supplementary Note 5). This is similar to the recently experimentally demonstrated lattice of unitarily coupled waveguides^28, 29^. Alternative circuits are waveguides arranged as a honeycomb and square lattice (Supplementary Figs. 3 and 4). The Lindblad operator for the honeycomb structure is $L_j = \mathop {\sum}\limits_{k = 1}^6 \left( { - 1} \right)^ka_{jk}$, where *j* indexes hexagonal cells and *k* numbers the modes in the cell. If each mode in a hexagonal cell has the same amplitude, the cell collectively constitutes a stationary, compacton-like state. These states satisfy $\left\langle {L_j} \right\rangle = 0$, ∀*j*. It can be noted that they are robust with respect to additional losses in modes neighbouring the cell. If there are some losses within the stationary cell itself, some non-vacuum states can still be supported (Supplementary Note 5). Moreover, coherence can spread diffusively in the lattice. Detailed discussion on the dissipative localisation in the diffusive square lattice can be found in Supplementary Note 5.

### Quantum thermodynamical interpretation

The coherent diffusive dynamics of DCC also have an intriguing quantum thermodynamical interpretation. For a long DCC with identical initial coherent states of modes, the system will strongly equalise any fluctuations. We then dissipatively couple one further ‘signal’ mode to this chain. This DCC will act as a reservoir, driving the signal mode towards some state independent of its initial excitation, asymptotically disentangled from the remainder of the chain. The state of the DCC after this interaction will belong to the same class of macrostates as initially. Thus the long DCC chain is acting as a catalytic reservoir to the signal mode, and forthwith we use the term reservoir to describe the arrangement.

Let us consider a DCC with $N + 1 \gg 1$ oscillators in coherent states, each of amplitude *α*, and the dissipatively connected signal mode having amplitude *a*_0_. For an arbitrary initial state of the *a*_0_ mode represented as $\rho _{a0} = \mathop {\sum}\limits_j p_j\left| \right.{\beta _j}\left. \right\rangle \left\langle \right.{\beta _j} \left. \right|_0$ ^30^, the stationary state of the chain will be $\rho _{\text{st}} = \mathop {\sum}\limits_j p_j\mathop {\prod}\limits_{k = 0}^{N + 1} \left| {\bar \alpha _j} \right\rangle \left\langle {\bar \alpha _j} \right|_k$, with $\bar \alpha _j = \frac{1}{{N + 2}}\left( {\beta + \left( {N + 1} \right)\alpha _j} \right)$. For any finite set of *β*_*j*_, the fidelity of the stationary state with the product of coherent states, $\left| {\mathrm{\Phi }} \right\rangle = \mathop {\prod}\limits_{j = 0}^{N + 1} \left| \alpha \right\rangle _j$, tends to unity for large *N*. A sufficiently long DCC will, therefore, evolve any signal state into the coherent state initialised on the other oscillators in the chain. Hence, the DCC is indeed acting as a reservoir, washing away any information about the initial state. However, this clearly happens at a certain cost. The energy difference between the initial and asymptotic states of the chain and mode *a*_0_ is given by:

4 $${\mathrm{\Delta }}E = \frac{{N + 1}}{{N + 2}}\mathop {\sum}\limits_j p_j\left| {\beta _j - \alpha } \right|^2.$$

Note that in the limit of large *N*, the energy balance of the mode *a*_0_ is the difference between the energies of the initial and the asymptotic state of the mode *a*_0_: ${\mathrm{\Delta }}E_0 \approx \mathop {\sum}\limits_j p_j\left| {\beta _j} \right|^2 - \left| \alpha \right|^2$. It can be equal to the energy loss of the whole mode *a*_0_ plus DCC system, which holds for signal states satisfying $\left| \alpha \right| = \mathop {\sum}\limits_j p_j\left| {\beta _j} \right|{\mathrm{cos}}\left\{ {{\mathrm{arg}}\left( {\beta _j} \right) - {\mathrm{arg}}(\alpha )} \right\}$.

Therefore, erasure of the state of mode *a*_0_ can be performed without energy change of our reservoir. Such an action seems to contradict the famous Landauer’s erasure principle: in order to erase information irreversibly, by an action of the environment, energy transfer into the environment needs to occur^31^. However, this principle was formulated for classical systems. The quantum Landauer’s principle holds under different assumptions. These consist of the reservoir being a closed, Gibbs state system, which is entirely uncorrelated with the signal state. If the reservoir is not isolated from the environment, the applicability of the Landauer’s principle is questionable^32^. Indeed, the use of an additional quantum system coupled to the reservoir allows the state of the signal to be erased without entropic change. The DCC is an example of the reservoir with such an additional quantum system.

## Discussions

In summary, we have illustrated intriguing possibilities for photonics that are generated by diffusive light propagation. The dissipative coupling of bosonic modes can allow light to flow like heat, whilst retaining coherence and even entanglement. A linear system of dissipatively coupled waveguides can act as an optical equaliser, smoothing fluctuations in amplitude and phase towards a common output. Any input state, classical or non-classical, will evolve into a completely symmetrised, correlated, state of the whole system. This equalising action has been experimentally demonstrated with coherent input to an elementary photonic circuit (Fig. 3) and for the chain of five waveguides (Fig. 4). Further, we have outlined dissipative circuits which can catalytically direct the flow of light across multiple channels, or even support stationary lattice states without impurity.

Generally, integrated waveguide networks lend themselves to applications in quantum information science^33^, particularly in quantum communication. Quantum communication based on so-called qumodes (continuous variables optical quantum systems) has already proven feasible in terms of point-to-point transmission of quantum states (see refs. ^34–36^) and this can be used in a number of applications such as quantum metrology and sensing, quantum cryptography and quantum signatures. Our coherent diffusive circuits are relevant as generic systems of qumodes propagating in integrated lossy networks where the loss mechanism provides quantum state engineering.

In the context of quantum thermodynamics, the DCC itself can be considered as a reservoir with non-trivial properties. Remarkably, the state of the reservoir can remain unchanged throughout the process of interaction with an external 'signal' mode and allow non-Landauer erasure to be performed. Further, the optical equalisation and quantum evolution towards a stationary state can be used to study equilibration and thermalisation processes in quantum theory, one of the central problems in quantum thermodynamics^37^. In the future, we believe that diffusive photonic systems will find practical application both in studying the fundamental processes of structurally engineered open systems and in an array of integrated photonic technologies. Furthermore, the non-linear DCC can be engineered and implemented for producing and distributing non-Gaussian states^38^.

## Methods

### Fabrication and characterisation of the photonic devices

The photonic devices formed by arrays of identical optical waveguides were fabricated using femtosecond laser writing technique^24^. A 30-mm-long borosilicate substrate (Corning Eagle^2000^) was mounted on *x*-*y*-*z* translation stages (ABL1000), and each waveguide was fabricated by translating the stages once through the focus of the fs laser pulses generated by an Yb-doped fibre laser (Menlo Systems, BlueCut; 350 fs, 500 kHz and 1030 nm). The waveguide arrays were characterised using single-mode-fibre input coupling and free-space output coupling. To excite waveguides with a tunable wavelength of light, a photonic crystal fibre^39^ was pumped using sub-picosecond laser pulses of 1064 nm wavelength to generate a broadband supercontinuum. A tunable monochromator placed after the supercontinuum source was used to select narrow band (~3 nm) light, which was coupled into an optical fibre (SMF-600). This fibre was then coupled to the desired waveguides. The output intensity distribution was observed using a CMOS camera (Thorlabs, DCC1545M).

### Data availability

Raw experimental data are available through Heriot-Watt University PURE research data management system (https://doi.org/10.17861/15c1715e-a7c4-4bbf-beb4-91341f1c5ca0).

## Electronic supplementary material

Supplementary Information

## References

[1] Palma GM, Suominen KA, Ekert AK (1996). Quantum computers and dissipation. Proc. R Soc. London Ser. A.

[2] Zanardi P, Rasetti M (1997). Noiseless quantum codes. Phys. Rev. Lett..

[3] Braun D (2002). Creation of entanglement by interaction with a common heat bath. Phys. Rev. Lett..

[4] Benatti F, Floreanini R, Piani M (2003). Environment induced entanglement in markovian dissipative dynamics. Phys. Rev. Lett..

[5] Lidar, D. A. & Whaley, K. B. in *Irreversible Quantum Dynamics*, Vol. 622 (eds Benatti, F. & Floreanini, R.) 83–120 (Springer, Berlin, 2003).

[6] Carvalho ARR, Milman P, de Matos Filho RL, Davidovich L (2001). Decoherence, pointer engineering, and quantum state protection. Phys. Rev. Lett..

[7] Man ZX, Xia YJ, Lo Franco R (2015). Cavity-based architecture to preserve quantum coherence and entanglement. Sci. Rep..

[8] Man ZX, Xia YJ, Lo Franco R (2015). Harnessing non-Markovian quantum memory by environmental coupling. Phys. Rev. A..

[9] Poyatos JF, Cirac JI, Zoller P (1996). Quantum reservoir engineering with laser cooled trapped ions. Phys. Rev. Lett..

[10] Ezaki H, Hanamura E, Yamamoto Y (1999). Generation of phase states by two-photon absorption. Phys. Rev. Lett..

[11] Clark S, Peng A, Gu M, Parkins S (2003). Unconditional preparation of entanglement between atoms in cascaded optical cavities. Phys. Rev. Lett..

[12] Verstraete F, Wolf MM, Ignacio Cirac J (2009). Quantum computation and quantum-state engineering driven by dissipation. Nat. Phys..

[13] Pastawski F, Clemente L, Cirac JI (2011). Quantum memories based on engineered dissipation. Phys. Rev. A..

[14] Diehl S, Rico E, Baranov MA, Zoller P (2011). Topology by dissipation in atomic quantum wires. Nat. Phys..

[15] Rudner MS, Levitov LS (2009). Topological transition in a non-hermitian quantum walk. Phys. Rev. Lett..

[16] Zeuner J (2015). Observation of a topological transition in the bulk of a non-hermitian system. Phys. Rev. Lett..

[17] Marandi A, Wang Z, Takata K, Byer RL, Yamamoto Y (2014). Network of time-multiplexed optical parametric oscillators as a coherent Ising machine. Nat. Photonics.

[18] Pal V, Tradonsky C, Chriki R, Friesem AA, Davidson N (2017). Observing dissipative topological defects with coupled lasers. Phys. Rev. Lett..

[19] Tradonsky C, Pal V, Chriki R, Davidson N, Friesem AA (2017). Talbot diffraction and Fourier filtering for phase locking an array of lasers. Appl. Opt..

[20] Rechtsman MC (2013). Photonic floquet topological insulators. Nature.

[21] Mukherjee S (2017). Experimental observation of anomalous topological edge modes in a slowly driven photonic lattice. Nat. Commun..

[22] Peruzzo A (2010). Quantum walks of correlated photons. Science.

[23] Guo A (2009). Observation of PT-symmetry breaking in complex optical potentials. Phys. Rev. Lett..

[24] Davis KM, Miura K, Sugimoto N, Hirao K (1996). Writing waveguides in glass with a femtosecond laser. Opt. Lett..

[25] Garanovich IL, Longhi S, Sukhorukov AA, Kivshar YS (2012). Light propagation and localization in modulated photonic lattices and waveguides. Phys. Rep..

[26] Biggerstaff DN (2016). Enhancing coherent transport in a photonic network using controllable decoherence. Nat. Commun..

[27] Mogilevtsev D (2015). Quantum tight-binding chains with dissipative coupling. New. J. Phys..

[28] Mukherjee S (2015). Observation of a localized flat-band state in a photonic lieb lattice. Phys. Rev. Lett..

[29] Vicencio RA (2015). Observation of localized states in lieb photonic lattices. Phys. Rev. Lett..

[30] Rehacek J, Mogilevtsev D, Hradil Z (2010). Operational tomography: fitting of data Patterns. Phys. Rev. Lett..

[31] Landauer R (1961). Irreversibility and heat generation in the computing process. IBM J. Res. Dev..

[32] Reeb D, Wolf MM (2014). An improved Landauer principle with finite-size corrections. New J. Phys..

[33] Brecht B, Reddy, Dileep V, Silberhorn C, Raymer MG (2015). Photon temporal modes: a complete framework for quantum information science. Phys. Rev. X.

[34] Grosshans F (2003). Quantum key distribution using gaussian-modulated coherent states. Nature.

[35] Croal C (2016). Free-space quantum signatures using heterodyne measurements. Phys. Rev. Lett..

[36] Guenthner K (2017). Quantum-limited measurements of optical signals from a geostationary satellite. Optica.

[37] Gogolin C, Eisert J (2016). Equilibration, thermalisation, and the emergence of statistical mechanics in closed quantum systems. Rep. Prog. Phys..

[38] Mogilevtsev D, Shchesnovich VS (2010). Single-photon generation by correlated loss in a three-core optical fiber. Opt. Lett..

[39] Stone JM, Knight JC (2008). Visibly “white” light generation in uniform photonic crystal fiber using a microchip laser. Opt. Express.

