# Supplementary material for: Dissipatively coupled waveguide networks for coherent diffusive photonics
Source: Nat Commun. 2017 Dec 4;8:1909. doi: 10.1038/s41467-017-02048-4 (PMC5712527; doi:10.1038/s41467-017-02048-4)
Supplement: Supplementary file 1 — Supplementary Information [file 41467_2017_2048_MOESM1_ESM.pdf]

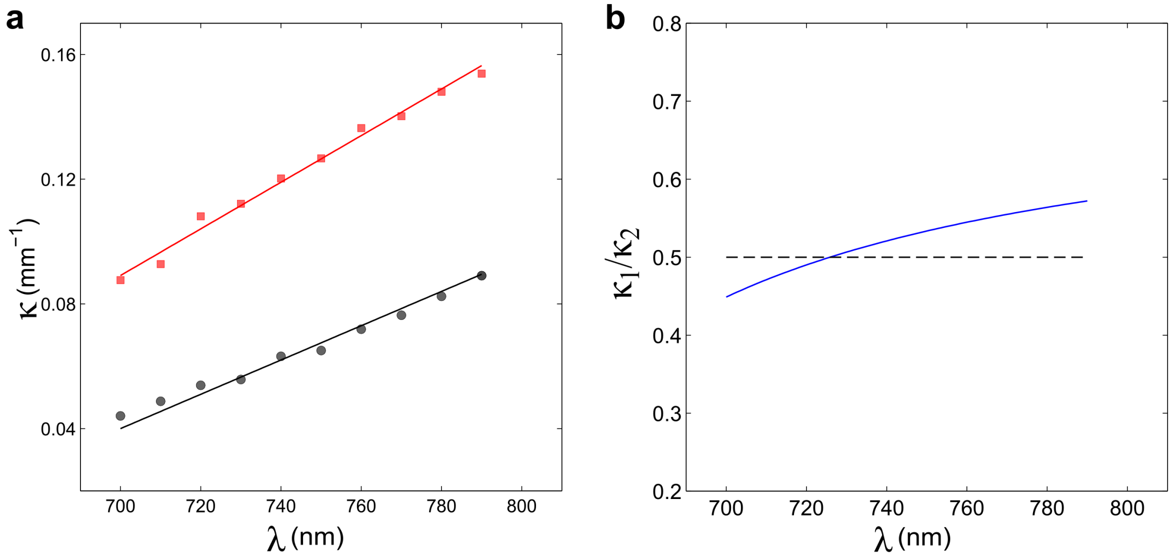

Supplementary Figure 1: **Measured variation of coupling strengths. a,** Variation of coupling constants ($\kappa_1 \rightarrow$ black and $\kappa_2 \rightarrow$ red) as a function of the wavelength of light. The solid lines are linear fits. **b,** Variation of κ_1/κ_2 as a the function of wavelength of light. In the wavelength range of interest (i.e. $700 - 790$ nm), this ratio remains very close to the desired value of 0.5 (dashed line) with a maximal deviation of $\approx \pm 0.05$.

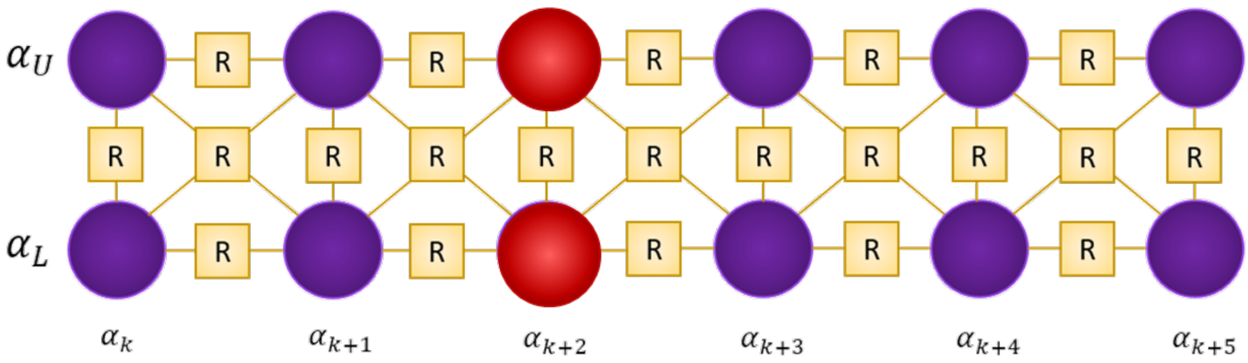

Supplementary Figure 2: **Schematic of a 2D diffusive photonic circuit (double chain).** The circuit consists of two parallel dissipatively coupled chains. Here, the squares represent reservoirs (R), the circles are bosonic modes (the red circles indicate possible initial excitations).

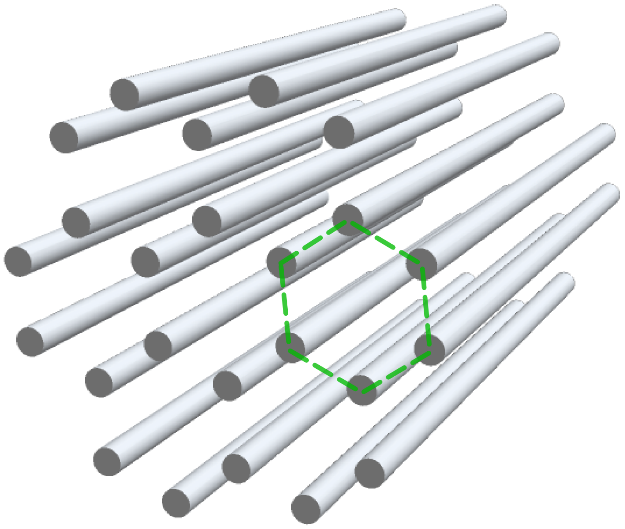

Supplementary Figure 3: **A diffusive photonic honeycomb lattice.** When all the bosonic modes in a hexagonal cell (indicated by the dashed line) have the same amplitude, the cell can support a stationary, compacton-like state.

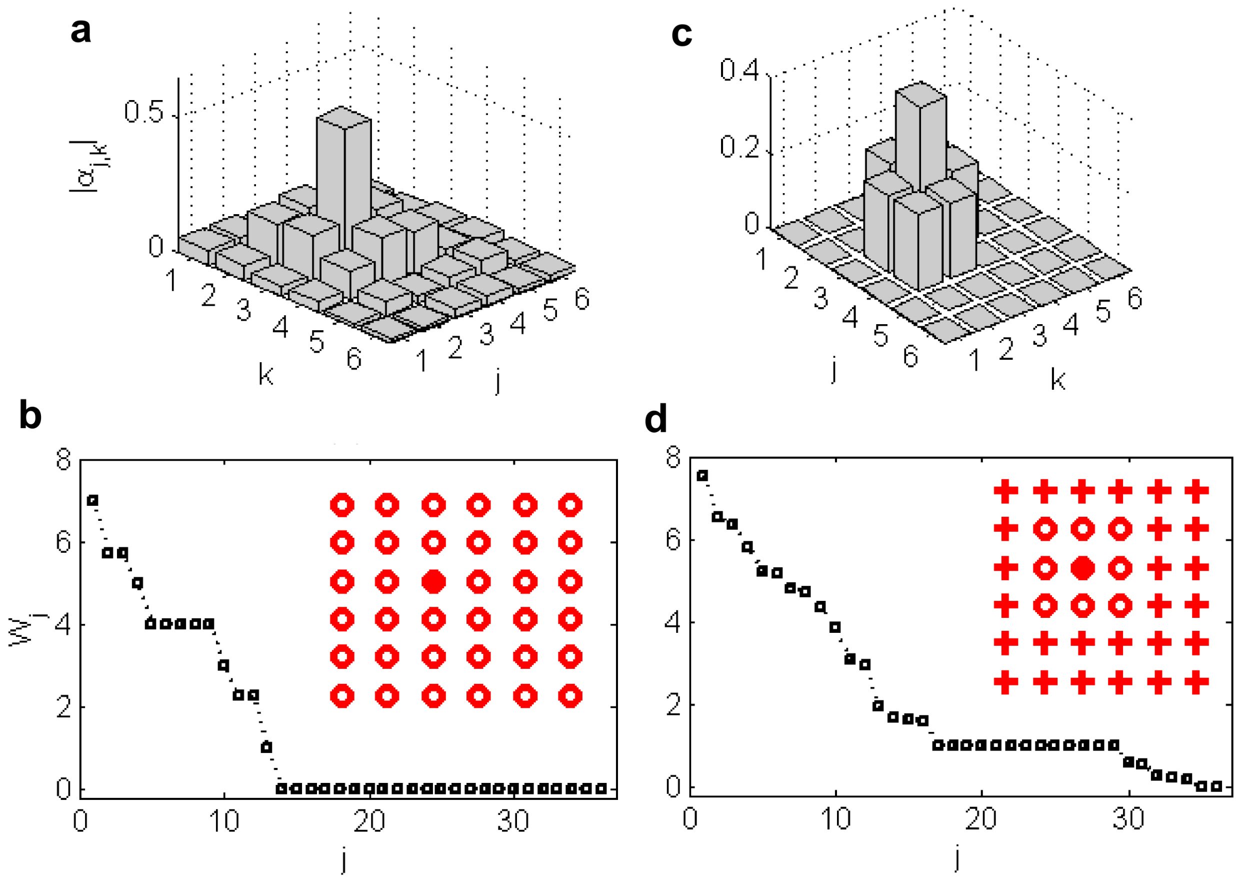

Supplementary Figure 4: **A diffusive square lattice.** Stationary distributions of absolute values of modal amplitudes for a 6×6 square lattice without (a) and with (c) additional losses at the sites indicated by red crosses in the inset of (d). Eigenvalues (in the units of γ) of the systems [Supplementary Eq. 18] without (b) and with (d) additional losses. Filled circles in the insets of (b, d) denote the initial excitations.

Supplementary Note 1: Experimental model

The coherent diffusive photonic circuits, considered in the main text, are described by the following generic quantum master equation:

$$\frac{d}{dt}\rho = \sum_{j=1}^{N} \gamma_j \left(2A_j \rho A_j^\dagger - \rho A_j^\dagger A_j - A_j^\dagger A_j \rho \right), \tag{1}$$

where $\rho(t)$ is the density matrix and A_j denote the Lindblad operators for mode j. Quantities γ_j are relaxation rates into corresponding reservoirs describing the coherence diffusion rate between neighbouring waveguides. Supplementary Eq. (1) with the Lindblad operator with $A_j = a_j - a_{j+1}$, where a_j ($a_j^\dagger$) is the bosonic annihilation (creation) operator, follows from the usual model of the unitary coupled tight-binding chain of linear waveguides with every second waveguide being subjected to strong loss. The details of the derivation can be found, for example, in ref. 1. This time evolution is modelled in the experiment by the arrays of coupled optical waveguides. The reservoirs are realised by mutually coupling each pair of waveguides to a linear chain of further waveguides as shown in Fig. 1 in the main text.

An interesting feature of the behaviour of light in these devices is the interchangeability between propagation distance and wavelength. The effective time of evolution γt can be altered both by changing the length of the waveguide block, or the wavelength of incident light. As the wavelength is tuned, κ_1 changes almost linearly to keep $\kappa_1/\kappa_2 \approx 0.5$, maintaining the correct character of dynamics. Notice that the dependence of diffusion rates, γ_j, on time changes neither the diffusive character of the dynamics nor the asymptotic state provided that always $\gamma_j(t) > 0$.

Supplementary Note 2: Measurement of evanescent coupling

As mentioned in the main text, the control of evanescent coupling is crucial for the experimental realisation of the diffusive equaliser. In Supplementary Fig. 1 we present the measured variation of $\kappa_{1,2}$ as a function of the wavelength of incident light, λ. We fabricated two types of directional couplers (each consisting of two evanescently coupled straight waveguides) which are the building blocks of the photonic circuits shown in Fig. 1 (main text). For the first type, where the two waveguides are at 45° angle, the coupling constant is κ_1 and that for the second type (consisting of two horizontally separated waveguides) is κ_2. Measuring the light intensities at the output of these 30-mm-long directional couplers, $\kappa_{1,2}(\lambda)$ were calculated [2].

It was observed that for these couplers, the ratio of $\kappa_{1,2}$ remains very close to the desired value of 0.5 with a maximum deviation of $\approx \pm 0.05$.

Supplementary Note 3: Dynamics of the dissipatively coupled bosonic chain

Due to the linearity of Supplementary Eq. (1), the initial coherent states propagation through DCC remain coherent states at any time moment of dynamics described by Supplementary Eq. (1). Consider the Glauber P-function for the density matrix, $\rho(t)$, of the state describing the circuit:

$$\rho(t) = \int d^2\vec{\alpha} P(\vec{\alpha}, \vec{\alpha^*}; t)|\vec{\alpha}\rangle\langle\vec{\alpha}|, \tag{2}$$

where $|\vec{\alpha}\rangle = \prod_j |\alpha_j\rangle$; $|\alpha_j\rangle$ is the coherent state of the j-th mode of the circuit and the amplitude α_j represents the j-th elements of the vector $\vec{\alpha}$. For the DCC with $N+1$ modes and jth Lindblad

operator represented as $A_j = a_j - a_{j+1}$, the solution for the P-function is obtained from the following Fokker-Planck equation:

$$\frac{\partial}{\partial t}P(\vec{\alpha},\vec{\alpha^*};t) = (\sum_{j=1}^{N}\gamma_j(\frac{\partial}{\partial\alpha_j}\alpha_j - \frac{\partial}{\partial\alpha_j}\alpha_{j+1} - \frac{\partial}{\partial\alpha_{j+1}}\alpha_j + \frac{\partial}{\partial\alpha_{j+1}}\alpha_{j+1}) + \text{h.c.})P(\vec{\alpha},\vec{\alpha^*};t) \quad (3)$$

Due to the linearity of this equation, the solution can be represented as $P(\vec{\alpha},\vec{\alpha^*};t) = P(\vec{\alpha}(t),\vec{\alpha^*}(t))$, where dynamics of amplitudes is described by Eq. (2) of the main text. It is instructive to represent the initial state in terms of discrete superposition of coherent state projectors [3]:

$$\rho(0) = \sum_{\forall k} p_k \prod_{\forall j} |\alpha_{jk}\rangle\langle\alpha_{jk}|_j, \quad (4)$$

where the index k labels a set of amplitudes $\{\alpha_{k1}, \alpha_{k2}\ldots\}$. The time-dependent Glauber function corresponding to the initial state [Supplementary Eq. (4)] is given by Supplementary Eq. (1) as

$$P(\vec{\alpha},\vec{\alpha^*};t) = \sum_{\forall k} p_k \prod_{\forall j} \delta(\alpha_j - \alpha_{jk}(t))\delta(\alpha_j^* - \alpha_{jk}^*(t)), \quad (5)$$

where amplitudes $\alpha_{jk}(t)$ for the DCC are defined from Eq. (2) of the main text.

As follows from Supplementary Eq. (1), any density matrix which is function of operators $A_{\text{sum}} = \sum_{j=1}^{N+1} \frac{a_j}{\sqrt{N+1}}$, $A_{\text{sum}}^\dagger$, and the vacuum, $\rho_{\text{vac}} = \prod_{\forall j} |0\rangle\langle 0|_j$, corresponds to a stationary state. These states can be of a quite different nature. The stationary state can be just the pure product of coherent states of individual modes with the same amplitude:

$$\rho_{\text{st}} = |\Phi_{\text{st}}\rangle\langle\Phi_{\text{st}}|, \quad |\Phi_{\text{st}}\rangle = \prod_{\forall j} |\alpha\rangle_j. \quad (6)$$

However, it can also be quite exotic, for example, it can be a Schrödinger-cat entangled state with $|\Phi_{\text{st}}\rangle \propto \sum_{k=1}^{K} w_k \prod_{\forall j} |\alpha_k\rangle_j$, where K is the number of different components in our cat-state and w_k are

scalar weights. The Gibbs state

$$\rho_{\text{st}} = \frac{\exp\{-\beta A_{\text{sum}}^{\dagger} A_{\text{sum}}\}}{\text{Tr}\{\exp\{-\beta A_{\text{sum}}^{\dagger} A_{\text{sum}}\}\}} \tag{7}$$

also belongs to the stationary states of the system. This state has maximal entropy for the given sum of the second-order coherences, $\langle a_k^{\dagger} a_l \rangle$ (which is also conserved by the dynamics). As was already mentioned, the stationary state can also be maximally entangled.

The DCC evolves toward a stationary state in a quite remarkable way. The initial state of the DCC with $N+1$ modes corresponding to the coherent state of all the chain modes, $|\Phi_0\rangle = \prod\limits_{\forall j} |\alpha_j\rangle_j$, evolves to the product of coherent states with equal amplitudes, $|\Phi_{t\to\infty}\rangle = \prod\limits_{\forall j} |\alpha_{\text{sum}}\rangle_j$, where the amplitude is the averaged sum of all the amplitudes, $\alpha_{\text{sum}} = \sum\limits_j \alpha_j/(N+1)$. Then an arbitrary initial state of the DCC [Supplementary Eq. (4)] will be asymptotically reduced to the following form:

$$\rho_{\text{st}} = \sum_{\forall k} p_k \prod_{j=1}^{N+1} |\bar{\alpha}_k\rangle\langle\bar{\alpha}_k|_j \tag{8}$$

with $\bar{\alpha}_k = \frac{1}{N+1} \sum\limits_{j=1}^{N+1} \alpha_{jk}$. Actually, the DCC drives the initial state to the symmetrical state over all the modes. Note, that the smoothing action of DCC is preserved even for the case of different decay rates, $\gamma_j \neq 0$. Stationary states do not depend on them.

Supplementary Note 4: Two-arm distributor structure

The prerequisite of the distributing action considered here is the existence of several localised stationary states of the structure described by the master equation, Supplementary Eq. (1). For the

sake of simplicity, we consider here pure stationary states. We call the state $|\chi^{\text{loc}}\rangle$ "localised" if exists some subset, $\{m\}$, of $M<K$ systems of our dissipatively coupled photonic circuit such that $\sum_{k\in\{m\}}\langle\chi^{\text{loc}}|a_k^\dagger a_k|\chi^{\text{loc}}\rangle>0$, whereas for systems out of the subset $\{m\}$ we have $\sum_{k\notin\{m\}}\langle\chi^{\text{loc}}|a_k^\dagger a_k|\chi^{\text{loc}}\rangle=0$. The most simple and obvious distributing action would be possible if the initial state of the structure, ρ_0, is orthogonal to some localised stationary state, $\langle\chi_j^{\text{loc}}|\rho_0|\chi_j^{\text{loc}}\rangle=0$. Then the part of the structure corresponding to subset $\{m_j\}$ will not be excited in the process of dynamics described by Supplementary Eq. (1). For such a distributor to be non-trivial, sets corresponding to different localised states, $\{m_j\}$, have to be partially overlapping. The distributor can be realised even in the case when the localised stationary states corresponding to different parts of the structure are not mutually orthogonal.

Let us illustrate our consideration with the example of the slightly modified DCC. For the structure depicted in Fig. 5a of the main text, modes in the arms are coupled pairwise, $A_j=a_j-a_{j+1}$ for $j=1\dots N-1, N+2\dots 2N$. For the central controlling node $L_N=a_N-a_R+a_L-a_{N+1}$. From the master equation, Supplementary Eq. (1), the equation similar to Eq. (2) of the main text can be obtained for each arm. For four modes of the central node the equations are as follows:

$$\frac{d}{dt}\alpha_N=-(\gamma_N+\gamma_{N-1})\alpha_N+\gamma_{N-1}\alpha_{N-1}+\gamma_N(\alpha_{N+1}+\alpha_H-\alpha_L), \tag{9}$$

$$\frac{d}{dt}\alpha_R=-\gamma_N(\alpha_R-\alpha_N+\alpha_{N+1}-\alpha_L), \tag{10}$$

$$\frac{d}{dt}\alpha_L=-\gamma_N(\alpha_L+\alpha_N-\alpha_{N+1}-\alpha_H), \tag{11}$$

$$\frac{d}{dt}\alpha_{N+1}=-(\gamma_N+\gamma_{N+1})\alpha_{N+1}+\gamma_{N+1}\alpha_{N+2}+\gamma_N(\alpha_N-\alpha_H+\alpha_L). \tag{12}$$

These equations describe 1D classical random walk. So, stationary states for arms decoupled from

the central node would be vectors with equal elements, $\alpha_j = \alpha$ for $j = 1 \ldots N$ or $j = N{+}1 \ldots 2N$ and arbitrary α. Also, there is a stationary state localised in two controlling modes, a_R and a_L, with $\alpha_R = \alpha_L$ and $\alpha_j = 0$, $j = 1 \ldots 2N$. Obviously, for the whole structure, the equal distribution of amplitudes in both arms $\alpha_j = \alpha$ for $j = 1 \ldots N$ and $j = N{+}1 \ldots 2N$, and equal amplitudes in the controlling modes, $\alpha_R = \alpha_L$ is also the stationary state. Excitation of just one arm and one of the controlling modes with equal amplitudes (i.e., for example, $\alpha_j = \alpha$ for $j = 1 \ldots N$, $\alpha_R = \alpha$ and $\alpha_R = 0$, $\alpha_j = 0$ for $j = N + 1 \ldots 2N$) is also a stationary state. By exciting control modes, a_R and a_L in certain states, one can make an initial excitation of a particular mode propagate either to the one arm, or to another, or to both arms simultaneously (see Fig. 5 in the main text). Notice, the such a distributing action can be achieved catalytically, since, as it follows from Supplementary Eq. (12), the coherence of two controlling modes are conserved, $\alpha_R(t) + \alpha_L(t) = \alpha_R(0) + \alpha_L(0)$, for any time-moment, t. In Fig. 5b, one can see an illustration of the distribution for the two-arm structure shown in Fig. 5a.

Supplementary Note 5: Double chain and dissipative localisation

For the sake of generalisation, now we consider two parallel dissipatively coupled chains as shown in Supplementary Fig. 2. The chain consists of squares, connected side by sides, so, the Lindblad operator of j-th square is

$$A_j = a_{j,+} - a_{j,-} + a_{j-1,+} - a_{j-1,-}. \tag{13}$$

We obtain the following set of equations for the coherent amplitudes:

$$\frac{d}{dt}\vec{\alpha}_j = -\gamma \hat{O}(2\vec{\alpha}_j - \vec{\alpha}_{j-1} - \vec{\alpha}_{j+1}), \tag{14}$$

where the matrix $\hat{O}$ has elements $\hat{O}_{j,k} = (-1)^{j+k}$, $j,k = 1,2$. The vector $\vec{\alpha}_j = [\alpha_{j,+}, \alpha_{j,-}]^T$. Despite being only a slight modification of the simplest DCC, the doubled chain has a number of drastically different features. First of all, any vector of coherent amplitudes, $\vec{\alpha}_j$, with equal upper (+) and lower (−) components is the stationary localised state. Then, initial excitation of any lattice site (say, $\alpha_{j,+} = x$) gives rise to the stationary state consisting of two-site localised state $\alpha_{j,+} = \alpha_{j,-} = x/2$ plus delocalised state $\alpha_{k,+} = (-1)^{j-k}x/2N$, $\alpha_{k,-} = (-1)^{j-k+1}x/2N$, where N is the number of systems in each chain. It is interesting that the double chain can serve as an analogous filter. If both the lower and upper chains are excited, the stationary result in each site would be half of the sum of the lower and upper initial amplitudes. Also, localised states are robust. Additional losses on sites out of the localisation region do not affect the localised states. However, they do affect the de-localised stationary states driving them to the vacuum.

Such localisation phenomena can hold also for infinite perfectly periodic dissipatively coupled photonic lattices. Let us assume Lindblad operators of the following form

$$A_j = \sum_{k \in \{n_j\}} x_{jk} a_k. \tag{15}$$

where $\{n_j\}$ denotes a set of modes coupled to the same dissipative reservoir; x_{jk} are scalar weights describing such a coupling. To avoid trivial localised states, we assume that there are no isolated sets, and for any $\{n_j\}$ there is a set $\{n_l\}$ such that the intersection, $\{n_j\} \cap \{n_l\}$, $j \neq l$, is not empty, but unequal to any of $\{n_j\}$. Additionally, for the ideally periodic structures, we assume that any operator, a_k, belongs to at least two different sets, and any set transforms to other set by translation along lattice vectors, $\vec{e}_i$. Obviously, for any localised stationary state we have $A_j \rho^{\text{loc}} = 0 \; \forall j$. From Supplementary Eq. (15) it follows that any localised state occupies at least two sites of the

structure. An example of the honeycomb lattice allowing for dissipative localisation is shown in Supplementary Fig. 3 and briefly discussed in the main text.

To demonstrate basic features of dissipative localisation, let us consider here a simple example of a square lattice (see insets in Supplementary Fig. 4). Denoting the sites in the upper left corner of each square as (j, k), we obtain the following Lindblad operators for such a lattice:

$$A_{j,k} = a_{j,k} + a_{j+1,k} + a_{j,k+1} + a_{j+1,k+1}. \tag{16}$$

The equations for the amplitudes, Supplementary Eqs. (1,16), then read:

$$\frac{d}{dt}\alpha_{j,k} = -\gamma_{j,k}\langle A_{j,k}\rangle - \gamma_{j+1,k+1}\langle A_{j+1,k+1}\rangle, \tag{17}$$

$$\frac{d}{dt}\alpha_{j+1,k} = -\gamma_{j,k}\langle A_{j,k}\rangle - \gamma_{j+1,k-1}\langle A_{j+1,k-1}\rangle. \tag{18}$$

As can be seen from Supplementary Eqs. (17, 18), the minimal localised states for an infinite square lattice of Supplementary Fig. 4 involve at least four sites (for example, the localised state can be in the set $\{m\} = \{(j+1,k),(j+2,k),(j+1,k+1),(j+2,k+1)\}$). An example of the localised state composed of coherent states is

$$|\Psi^{\text{loc}}\rangle = |\alpha\rangle_{j+1,k}|-\alpha\rangle_{j+2,k}$$
$$|\alpha\rangle_{j+2,k+1}|-\alpha\rangle_{j+1,k+1}\prod_{j,k\notin\{m\}}|0\rangle_{j,k}. \tag{19}$$

Any closed contour including either 0, 2 or 4 sites of every square can host a localised state. A finite lattice can also support localised edge states with even, as well as odd, number of sites. For example, the three-site edge state in the upper left corner of the lattice shown in the inset of Supplementary Fig. 4 can have the coherent state with amplitudes 2α in $(1,1)$ and states with the amplitudes $-\alpha$ in sites $(2,1)$ and $(1,2)$.

Localised states of a dissipatively coupled lattice can be arbitrarily extended. A state can propagate through the lattice exciting localised states in several cells. To illustrate the basic features of such propagation, let us consider the dynamics of just a single unit cell of the square lattice [just one $A_{j,k}$ of Supplementary Eq. (16)]. One has

$$\vec{a}(t \to \infty) = (1 - \mathbf{S}/4)\vec{a}(0), \tag{20}$$

where the vector of time-dependent modal amplitudes is $\vec{a}(t) = [\alpha_{1,1}(t), \alpha_{1,2}(t), \alpha_{2,1}(t), \alpha_{2,2}(t)]^T$ and $\mathbf{S}$ is the matrix of units. Supplementary Eq. (20) shows that the final result is an initial state minus the result of complete symmetrisation of it over the cell. A similar process occurs for the complete lattice. Symmetrical parts propagate. Curiously, this process is described by the classical two-dimensional random walk. Let us introduce variables $\lambda_{m,n}(t) = (-1)^{m+n}\langle A_{m,n}(t)\rangle$. For $\gamma_j \equiv \gamma > 0, \forall j$. From Supplementary Eqs. (17, 18) it follows that

$$\frac{d}{dt}\lambda_{m,n} = -4\gamma\lambda_{m,n} + \gamma\big(\lambda_{m+1,n} + \lambda_{m-1,n} + \lambda_{m,n-1} + \lambda_{m,n+1}\big). \tag{21}$$

Similar heat-like propagation of coherences was found recently in dissipatively coupled 1D spin chains [1]. An illustration of the stationary distribution arising from the initial excitation of just one mode is given in Supplementary Fig. 4a. In Supplementary Fig. 4b, a spectrum of the equation matrix for Supplementary Eqs. (17, 18) is given. The plateau of zero eigenvalues is separated from the non-zero eigenvalues with the gap of γ. Supplementary Eq. (21) points also to the existence of delocalised stationary modes given by the condition $\langle A_{m,n}\rangle = (-1)^{m+n}\alpha$.

Despite coupling to neighbour sites, the stationary localised state is completely impervious to additional loss even on sites adjacent to those where the stationary state is localised. It can be

seen even for the simplest example of the single-cell system. Taking the equation matrix Eq. (18) with two sites subjected to additional loss with the rate γ as $-\gamma(\mathbf{S} - \mathrm{diag}(1, 1, 0, 0))$, one gets $\vec{a}(t \to \infty) = \mathbf{O}\vec{a}(0)$, where the only non-zero elements of the matrix $\mathbf{O}$ are $O_{3,3} = O_{4,4} = 0.5$ and $O_{3,4} = O_{4,3} = -0.5$. Again, the result is the initial vector minus its symmetrisation, but only for the modes untouched by the additional loss. A similar effect holds for a larger lattice. In Supplementary Fig. 4c one can see an example of a localised state in the region free of additional loss arising from the initial excitation of just one initial mode. The inset in the Supplementary Fig. 4d shows sites affected by additional individual loss with rate γ. Supplementary Fig. 4d shows eigenvalues of the equation matrix Eq. (17, 18) for this case. Only two localised states survive for the case, and the gap between the zero plateau and decaying modes are closed; there are modes with decay rates much less than γ.

Naturally, the localised stationary state can be entangled. The simplest example of the entangled states for the minimal localised states of the infinite square lattice of Supplementary Fig. 4a up to the normalization factor is

$$\begin{aligned} |\Psi^{\mathrm{loc}}\rangle &= |\alpha\rangle_{j+1,k}|-\alpha\rangle_{j+2,k}|\alpha\rangle_{j+2,k+1}|-\alpha\rangle_{j+1,k+1} \\ &+ |-\alpha\rangle_{j+1,k}|\alpha\rangle_{j+2,k}|-\alpha\rangle_{j+2,k+1}|\alpha\rangle_{j+1,k+1} \end{aligned} \tag{22}$$

which for $|\alpha| > 0$ is entangled since an averaging over any mode included in this equation gives a mixed state. Up to the normalization factor, the reduced state of any three modes is given by

$$\rho_3 = |\psi_3^+\rangle\langle\psi_3^+| + |\psi_3^-\rangle\langle\psi_3^-| + e^{-2|\alpha|^2}(|\psi_3^+\rangle\langle\psi_3^-| + |\psi_3^-\rangle\langle\psi_3^+|), \tag{23}$$

where $|\psi_3^{\pm}\rangle = |\pm\alpha\rangle_l|\mp\alpha\rangle_m|\pm\alpha\rangle_n$, and indexes l, m, n number three modes remained after

averaging over the fourth one.

Another example of the localised state is the state with just a single photon distributed over several lattice sites, such as

$$|\Psi^{\mathrm{loc}}\rangle = \sum_{k\in\{n\}} (-1)^l |1\rangle_k, \tag{24}$$

where the index l numbers modes along the closed contour connecting all the sites belonging to the set $\{n\}$ where the state is localised; the state $|1\rangle_k$ corresponds to the photon in k-th mode and the vacuum in all other modes. The same localisation regions as for the coherent modal states are possible for both the perfect and finite lattices. For example, the upper-left corner state of the lattice presented in Supplementary Fig. **4**a is $|\Psi^{\mathrm{loc}}\rangle = 2|1\rangle_1 - |1\rangle_2 - |1\rangle_3$. The states of Supplementary Eq. (24) are entangled. Amount of entanglement is proportional to the number of systems in the localised state. For example, the generalised Schmidt number for the state of Supplementary Eq. (24) is $2(N-1)$, N being the number of sites [4]. Notice that existence of the localised state, Supplementary Eq. (24), points to the possibility of dissipative compacton-like localisation in fermionic lattices, too.

Supplementary References

1. Mogilevtsev, D., Slepyan, G. Ya., Garusov, E., Kilin, S. & Korolkova, N. Quantum tight-binding chains with dissipative coupling. *New J. Phys.* **17,** 043065 (2015).

2. Szameit, A., Dreisow, F., Pertsch, T., Nolte, S. & Tünnermann, A. Control of directional evanescent coupling in fs laser written waveguides. *Opt. Express* **15** (4), 1579-1587 (2007).

3. Rehacek, J., Mogilevtsev, D. & Hradil, Z. Operational Tomography: Fitting of Data Patterns. *Phys. Rev. Lett.* **105,** 010402 (2010).

4. Guo, Y. & Fan, H. A generalization of Schmidt number for multipartite states. *Int. J. Quantum Inform.* **13,** 1550025 (2015).
